# Enhancing Diagnostic Pathways for Bronchopulmonary Neuroendocrine Tumors: Assessment of the South Wales Neuroendocrine Tumor Service Transformation

**DOI:** 10.1111/1759-7714.70282

**Published:** 2026-04-20

**Authors:** Arouba Imtiaz, Oliver Burbidge, Mat Jones, Robin Ghosal, Janardhan Navaratnam, Craig Dyer, Helen E. Davies, Mohid S. Khan

**Affiliations:** ^1^ South Wales Neuroendocrine Cancer Service, Department of Gastroenterology University Hospital of Wales Cardiff UK; ^2^ Department of Respiratory Medicine Cardiff and Vale University Health Board Cardiff UK; ^3^ Department of Respiratory Medicine Nevill Hall Hospital Abergavenny UK; ^4^ Department of Respiratory Medicine Prince Philip Hospital Llanelli UK

## Abstract

**Background:**

Bronchopulmonary neuroendocrine tumors (bpNETs) are uncommon lung neoplasms posing significant diagnostic and therapeutic challenges. This study evaluates the impact of the transformation of the South Wales Neuroendocrine Cancer Service on diagnostic and management outcomes for bpNETs.

**Methods:**

We retrospectively analyzed data from patients with typical and atypical carcinoids (TC and AC) diagnosed before and after commissioned service transformation (September 2017). Data were collected from network cancer databases and clinical portals. Demographics, diagnostic pathway times, diagnostic tests, treatment modalities, and survival outcomes were analyzed.

**Results:**

Following transformation, significant reductions were observed in median times from presentation to diagnosis (*p* = 0.009), symptom onset to diagnosis (*p* = 0.006), and presentation to treatment (*p* = 0.020). No other significant differences were noted between pre‐ and post‐transformation groups. Incidental diagnoses increased, especially in TCs (53%). Usage of CgA, HIAA, EBUS biopsies, and Gallium PET scans increased post‐transformation. Surgical treatments were common, but there was an increase in systemic therapy post‐transformation.

**Conclusion:**

Implementation of a collaborative care model and NET service transformation led to significant improvements in pathway times. This is one of the few studies describing such improvements in NETs. While survival outcomes showed promising trends, further research is needed to assess the long‐term impact on patient outcomes.

## Graphical abstract

Implementation of a dedicated neuroendocrine cancer service in South Wales significantly shortened diagnostic and treatment timelines for bronchopulmonary NETs, demonstrating the benefits of coordinated specialist input.

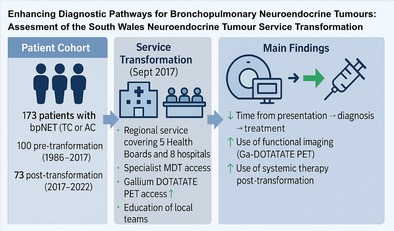

## 1 Introduction

Bronchopulmonary neuroendocrine neoplasms (NENs) are uncommon malignancies with an incidence of 0.2–2 per 100 000 [1]. These tumors account for approximately 25% of all lung cancers, comprising 20% small cell carcinoma, 3% large cell carcinoma, 1.8% typical carcinoids (TC), and 0.2% atypical carcinoids (AC), the latter two also known as neuroendocrine tumors (NETs) [2]. Although grouped together, the World Health Organization classifies bronchopulmonary NETs (bpNETs) into two categories based on histopathology review, mitotic rate, and necrosis, and although not mandated, Ki‐67 expression, as mentioned in European NET Society guidelines for NETs from the gastrointestinal tract [1, 3]. Well‐differentiated tumors include TC and AC, while poorly differentiated tumors include small and large cell carcinomas [3] with major clinical, epidemiologic, histologic, and genetic differences between the two categories. Notably, TC exhibits a higher 5‐year survival rate, ranging from 87% to 94%, compared to small cell lung cancer of 14.3% and large cell lung cancer of 9.6% [4, 5]. In this article, we consider only well‐differentiated TCs and ACs of the lung, which we will refer to as bpNET.

Bronchopulmonary NETs present with diverse clinical manifestations, influenced by factors such as tumor localization, size, type, and grade and stage [1]. The diagnosis of these tumors can be challenging due to the presence of nonspecific symptoms that often resemble common respiratory conditions [2]. Studies found that 5%–27% of patients presented with a cough, 11.6% with dyspnoea, 41%–49% experienced recurrent pneumonia, 23%–32% had hemoptysis, and 17%–39% were diagnosed incidentally [6, 7]. Moreover, certain types of NETs can produce ectopic hormones, leading to the development of distinct syndromes such as Cushing's syndrome (caused by excess adrenocorticotrophic hormone production) and carcinoid syndrome (caused by serotonin hormone production) [1]. These factors contribute to the complexity and potential delay in diagnosis of bpNETs.

Symptoms and routes of diagnosis in bpNETs have been described in several observational studies; however, detail on time to specialist referral relies predominantly on feedback from patient surveys rather than on objective measures. To enhance the diagnosis and management of all NETs, a transformation of the South Wales NET service was undertaken in September 2017. The new model incorporated consultant‐led NET clinics, regular multidisciplinary team (MDT) meetings, increased communication involving trained cancer nurse specialists, and, notably, collaborative relationships between the new NET team and local respiratory and oncology colleagues. The objective of this service evaluation was to assess the impact of this approach on the diagnosis and treatment of bpNETs, as well as to identify any areas that may require further improvement.

The objective of this research was to explore the routes of diagnosis, investigation, and treatment approaches for typical and atypical bpNETs within the patient population of South Wales. Additionally, the study aimed to assess potential temporal differences following the implementation of the revised South Wales NET Service in September 2017.

## 2 Methods

Patients with a histologically confirmed bpNET (TC or AC) were identified from NET and Health Board lung cancer databases in South Wales. Patients with small or large cell neuroendocrine carcinoma were excluded. The service evaluation was registered and approved by the Health Board's Service Improvement Committee and Information and Technology department. Cases were categorized according to whether the diagnosis was prior to or after the service transformation (August 31, 2017).

The transformation of the service was multifaceted and initially aimed at improving care for gastroenteropancreatic (GEP)‐NETs through national commissioning at a population level. This service transformation comprised several core components designed to streamline and improve care for patients with neuroendocrine tumors. A centralized digital referral system was introduced, enabling direct referrals to a small group of expert clinicians who led the neuroendocrine tumor multidisciplinary team (NET MDT). Referrals were triaged rapidly, allowing timely virtual input to local teams and ensuring efficient allocation of cases to MDT discussions. The frequency of NET MDT meetings increased from once to twice monthly, and a dedicated NET clinical nurse specialist (CNS) was appointed to support coordination of care and patient communication. Additional interventions included structured education for local teams, integration of bpNETs into the National Single Cancer Pathway, and improved access to specialist imaging (e.g., Gallium‐68 DOTATATE PET‐CT) and systemic therapy. MDT outcomes and clinical advice were documented on the Welsh Clinical Portal (WCP), accessible through all hospitals in Wales, improving transparency and follow‐up. Pre‐transformation, care was more fragmented, with variable access to MDT input and inconsistent communication between local and central teams. The new model aimed to create a more cohesive, equitable, and specialist‐led diagnostic and treatment pathway across South Wales.

Data were retrospectively collated from electronic medical records using the Welsh and Cardiff and Vale Clinical Portals incorporating hospital letters, MDT minutes, histological reports, and biochemical results. The duration of symptoms was obtained from clinical letters. The date of presentation was defined as the initial clinical encounter in primary care or first assessment of patients by a non‐respiratory hospital specialist prior to referral to the diagnosing respiratory team. If the tumor was incidental (asymptomatic patient), the date of the CT scan was considered as the date of presentation.

The date of confirmed diagnosis was defined as the time of tissue sampling, which confirmed the histological tumor type. All cases were reviewed by a dedicated NET MDT pathologist. Metastatic disease at diagnosis was determined radiologically based on CT, MRI, and PET scans (Gallium‐68 DOTATATE and FDG‐PET) imaging and confirmed following the consensus agreement of a central NET MDT team. Tumor classification and grading were conducted according to the World Health Organization criteria (2004; as proposed by the European Neuroendocrine Tumor Society (ENETS) guidelines) and the eighth edition of the Union for International Cancer Control/American Joint Committee on Cancer (UICC/AJCC) TNM classification [8, 9]. Carcinoid syndrome was clinically diagnosed in patients with elevated urinary 5HIAA levels (> 60 μmol/24 h) and symptoms of flushing and/or diarrhea. An elevated chromogranin A level was defined as being greater than 120 pmol/L, twice the upper limit of normal. Cushing's syndrome was diagnosed based on clinical symptoms and elevated levels of adrenocorticotrophic hormone. Patients with multiple endocrine neoplasia 1 (MEN1) syndrome were diagnosed based on confirmatory genetic testing.

Tumor recurrence was assessed in all postoperative patients and was diagnosed radiologically. Systemic treatment for locally advanced and metastatic bpNET, with chemotherapy, somatostatin analogues, and/or targeted therapies, was considered on a case‐by‐case basis with decisions determined following discussion at the central NET multidisciplinary meeting. Treatments were given in line with European guidelines, including ENETS and European Society for Medical Oncology (ESMO) [8, 10].

Data collected were analyzed using IBM SPSS Statistics for Windows, version 27. Statistical analysis involved chi‐squared test for categorical variables and Mann–Whitney *U* test for continuous variables, with statistical significance set at *p* < 0.05.

## 3 Results

This study identified a cohort of 173 patients diagnosed with well‐differentiated bpNETs (TC or AC). Seventy‐three patients were diagnosed after the transformation of the service (September 2017 to December 2022) and 100 were diagnosed prior to service transformation (June 1996 to August 2017). Table 1 demonstrates demographic and clinical data for both pre‐transformation and post‐transformation groups.

**TABLE 1:** Patient demographics and clinical data.

	Total patients (%) *n* = 173	Pre‐transformation group (%) *n* = 100	Post‐transformation group (%) *n* = 73	*p*
Sex, number				
Male	64 (37)	37 (37)	27 (37)	0.999
Female	109 (63)	63 (63)	46 (63)	
Median age, years (range)	68 (24–91)	66 (24–91)	70 (28–86)	0.285
Type of bpNET, number				
Typical	120 (69)	72 (72)	48 (66)	0.378
Atypical	53 (31)	28 (28)	25 (34)	
Functional status, number				
Non‐functioning	163 (94)	97 (97)	66 (90)	0.319
Carcinoid syndrome	8 (5)	3 (3)	5 (7)	
Cushing's syndrome	2 (1)	0 (0)	2 (3)	
Ki‐67 (%)				
Median	2.9	4.9	2.45	0.342
Range	0.9–50	0.9–50	0.9–40	
Metastatic disease at diagnosis				
M0	106 (61)	63 (63)	43 (59)	0.487
M1	59 (34)	34 (34)	29 (40)	
Unknown	4 (2)	3 (3)	1 (1)	
Location of metastases				
Bone	10 (17)	4 (12)	6 (22)	0.438
Liver	20 (33)	11 (33)	9 (33)	
Other	7 (12)	2 (6)	5 (19)	
Stage				
I	66 (46)	37 (49)	29 (43)	0.411
II	7 (5)	2 (3)	5 (7)	
III	6 (4)	2 (3)	4 (6)	
IV	63 (44)	34 (45)	29 (43)	
Unknown	31	25	6	
Presence of MEN1	5 (3)	4 (4)	1 (1)	0.308
Presence of DIPNECH	14 (8)	5 (5)	9 (12)	0.081

Abbreviations: bpNET, bronchopulmonary neuroendocrine tumor; DIPNECH, diffuse idiopathic pulmonary neuroendocrine cell hyperplasia; MEN1, multiple endocrine neoplasia type 1.

**FIGURE 1:**
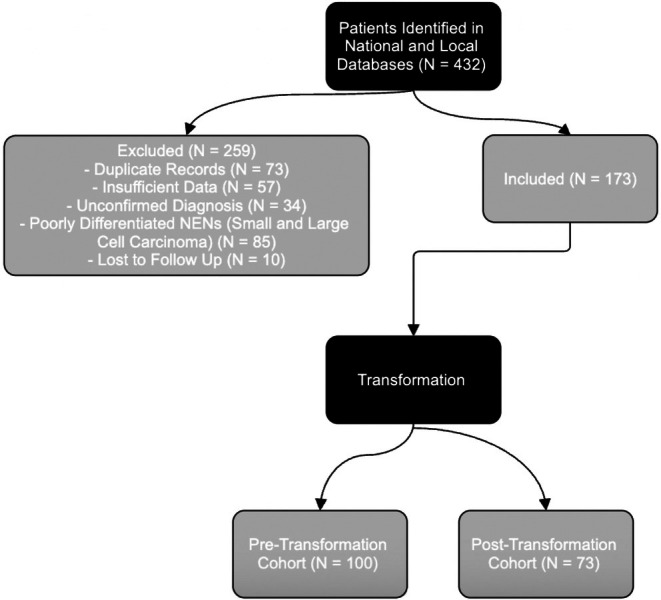
Flow chart showing patient inclusion.

Figure 1 shows the flow of patient inclusion. All patients with histologically confirmed well‐differentiated bronchopulmonary neuroendocrine tumors (typical or atypical carcinoid) were identified from the national Wales Cancer Network registry and local lung cancer databases. Exclusions included patients with poorly differentiated neuroendocrine carcinomas (*n* = 85), insufficient clinical or histological data (*n* = 57), lost to follow‐up (*n* = 10), duplicate records (*n* = 73), and unconfirmed diagnoses (*n* = 34). A total of 173 patients were included in the final analysis, with 100 in the pre‐transformation group and 73 in the post‐transformation group.

The incidence of metastatic disease was significantly higher in patients with ACs (58% vs. 23%, *p* = 0.000073). Only 6% of all cases had a functional secretory syndrome. MEN1 was present in 3% of cases, while diffuse idiopathic pulmonary neuroendocrine cell hyperplasia (DIPNECH) was present in 8%.

The majority of patients (88%) presented to the local respiratory team, followed by emergency medicine (9%), breast surgery (1%), endocrinology (0.5%), ENT (0.5%), general surgery (0.5%), rheumatology (0.5%), and urology (0.5%). Over half of the patients (53%) presented incidentally with no difference seen between the post‐transformation group and pre‐transformation cohorts (*p* = 0.094). Fifty‐seven percent of TCs were diagnosed incidentally, compared to 45% of ACs, a difference which was statistically significant (*p =* 0.034).

**FIGURE 2:**
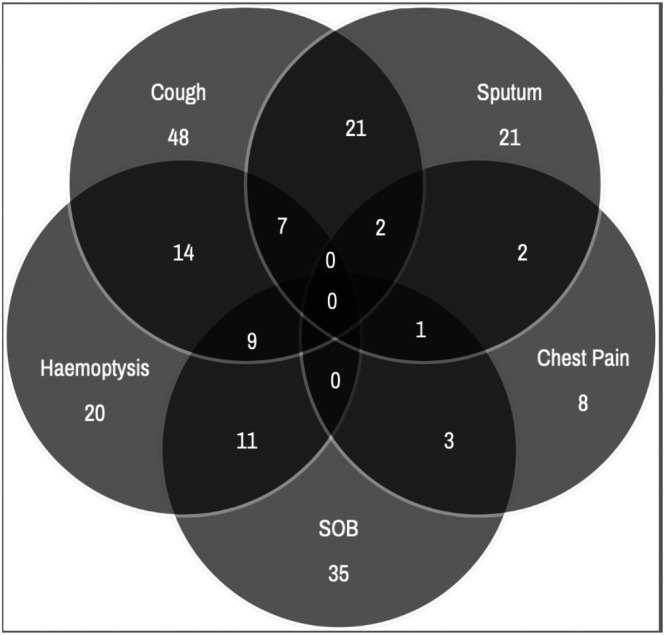
Venn diagram demonstrating the distribution and overlap of presenting symptoms (numbers of patients).

Surveillance imaging of a co‐existing malignancy represented the most common indication for undergoing a CT scan which identified the incidental presence of bpNETs, for example, bowel cancer surveillance accounted for the majority (*n* = 32, 35%). Additionally, 17 (19%) had imaging performed for abdominal symptoms, and 17 (19%) for musculoskeletal pain. Figure 2 illustrates the presenting symptoms in the total cohort. Cough was the most frequent symptom (28%), followed by shortness of breath (20%), hemoptysis (12%), sputum production (12%), and chest pain (5%). Ninety percent were polysymptomatic.

**FIGURE 3:**
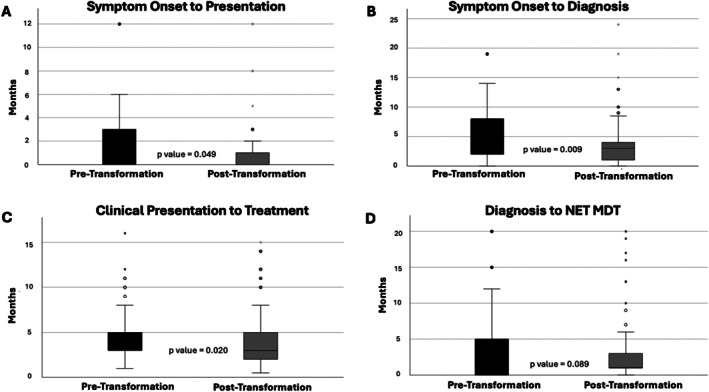
Box plots comparing pre‐ and post‐ transformation patient pathway metrics. (A) Time from symptom onset to presentation; (B) time from symptom onset to diagnosis; (C) time from clinical presentation to treatment; (D) time from diagnosis to NET MDT meeting. An asterisk denotes an extreme outlier (three times the interquartile range), circle represents other outliers (between 1.5 and 3 times the interquartile range).

Figure 3 illustrates the time intervals of diagnostic and treatment pathways in the pre‐ and post‐transformation groups. Overall, there was a reduction in most pathway times in the post‐transformation group including presentation to diagnosis (*p* = 0.009), to treatment (*p* = 0.020), and to MDT discussion (*p* = 0.025). Symptom‐to‐presentation times appeared to be similar between groups (*p* = 0.049). There was a reduction in the variation of these times after transformation.

During investigation, unsurprisingly, computerized tomography (CT) was central to diagnosis (Table 2). Since transformation, there was a significant reduction in the first pathological diagnosis of bpNET relying on surgical resection (60% vs. 40%, *p* = 0.008) with an increase in diagnostic biopsy (bronchoscopy, endobronchial ultrasound, or CT‐guided biopsy). More Gallium DOTATATE PET scans were performed after transformation (37%) compared to pre‐transformation (10%) (*p* < 0.001). Combined with octreotide scans, functional nuclear medicine imaging was conducted in 52% of cases post‐transformation (vs. 16% pre‐). FDG‐PET scans were undertaken in 58% of cases as part of lung cancer diagnostics prior to identification as a bpNET. Although a small proportion of cases had biomarker evaluation, chromogranin A was tested more in the post‐transformation group (*p* = 0.011), but elevated chromogranin levels were only noted in 3% of cases overall. Urinary 5HIAA levels were raised in only 5% in the total cohort.

**TABLE 2:** Diagnostic investigations performed.

	Total patients (%) *n* = 173	Pre‐transformation group (%) *n* = 100	Post‐transformation group (%) *n* = 73
Histological specimen diagnosing bpNET			
Surgical resection	89 (51)	60 (60)	29 (40)
CT‐guided biopsy	31 (18)	16 (16)	15 (21)
US‐guided liver biopsy	1 (1)	0 (0)	1 (1)
Bronchoscopy biopsy/EBUS sampling	52 (30)	24 (24)	28 (38)
CT scan			
Performed	165 (95)	92 (92)	73 (100)
Not performed	0 (0)	0 (0)	0 (0)
Unknown	8 (5)	8 (8)	0 (0)
FDG‐PET scan			
Performed	101 (58)	57 (57)	44 (60)
Not performed	64 (37)	35 (35)	29 (40)
Unknown	8 (5)	8 (8)	0 (0)
Octreotide scan			
Performed	27 (16)	16 (16)	11 (15)
Not performed	138 (80)	76 (76)	62 (85)
Unknown	8 (4)	8 (8)	0 (0)
Gallium PET			
Performed	35 (20)	10 (10)	27 (37)
Preoperatively	15 (9)	4 (4)	11 (15)
Postoperatively	7 (3)	2 (2)	6 (8)
Nonoperative	13 (8)	4 (4)	10 (14)
Not performed	130 (75)	82 (82)	46 (63)
Unknown	8 (5)	8 (8)	0 (0)
Chromogranin A			
Performed	62 (36)	28 (28)	34 (47)
High	5 (3)	0 (0)	5 (7)
Normal	57 (33)	28 (28)	29 (40)
Not performed	111 (64)	72 (72)	39 (53)
24 h Urinary 5‐HIAA			
Performed	58 (34)	30 (30)	28 (38)
Above 2× ULN	7 (5)	1 (1)	6 (8)
Below 2× ULN	51 (29)	29 (29)	22 (30)
Not performed	115 (66)	70 (70)	45 (62)

Abbreviations: 24 h 5HIAA, 24‐h 5‐hydroxy indoleacetic acid; bpNET, bronchopulmonary neuroendocrine tumor; CT, computed tomography; EBUS, endobronchial ultrasound; FDG, 18 fludeoxyglucose; PET, positron emission tomography; ULN, upper limit of normal; US, ultrasound.

Table 3 highlights the surgical treatments undertaken in 77% of patients. It is noteworthy that some patients were diagnosed with NETs postoperatively despite clinical suspicion of non‐NET malignancy prior to intervention (with no difference between TC and AC). Although this proportion reduced after transformation with an increase in diagnostic biopsy, there were no significant differences in types of surgical treatments undertaken between pre‐ and post‐transformation groups (with no difference between TC and AC).

**TABLE 3:** First‐line treatment.

Treatment	Total patients (%) *n* = 173	Pre‐transformation group (%) *n* = 100	Post‐transformation group (%) *n* = 73
Surgery	134 (77)	80 (80)	54 (74)
Lobectomy	93 (54)	55 (55)	38 (52)
Wedge resection	14 (8)	8 (8)	6 (8)
Bilobectomy	8 (5)	6 (6)	2 (3)
Segmentectomy	7 (4)	5 (5)	2 (3)
Endobronchial resection	5 (3)	2 (2)	3 (4)
Pneumonectomy	4 (2)	3 (3)	1 (2)
Sleeve resection	2 (1)	1 (1)	1 (2)
Somatostatin analogues	13 (8)	4 (4)	9 (12)
Advanced treatment (chemotherapy, everolimus, PRRT)	6 (3)	1 (1)	5 (7)
Radiotherapy	5 (3)	3 (3)	2 (3)
Watchful Waiting	15 (9)	12 (12)	3 (4)

Abbreviation: PRRT, peptide receptor radionuclide therapy.

A small number of patients underwent systemic therapy for advanced, recurrent, or metastatic disease (10%) (Table 3). Although numbers were small, fewer patients adopted a “watchful waiting” approach since transformation; 8% received somatostatin analogues (SSA), 3% were treated with alternative systemic treatments (including chemotherapy, e.g., everolimus); the latter number has risen since service transformation. Peptide receptor radionuclide therapy (PRRT) was administered in 3% of cases, with an equal distribution between both groups.

**FIGURE 4:**
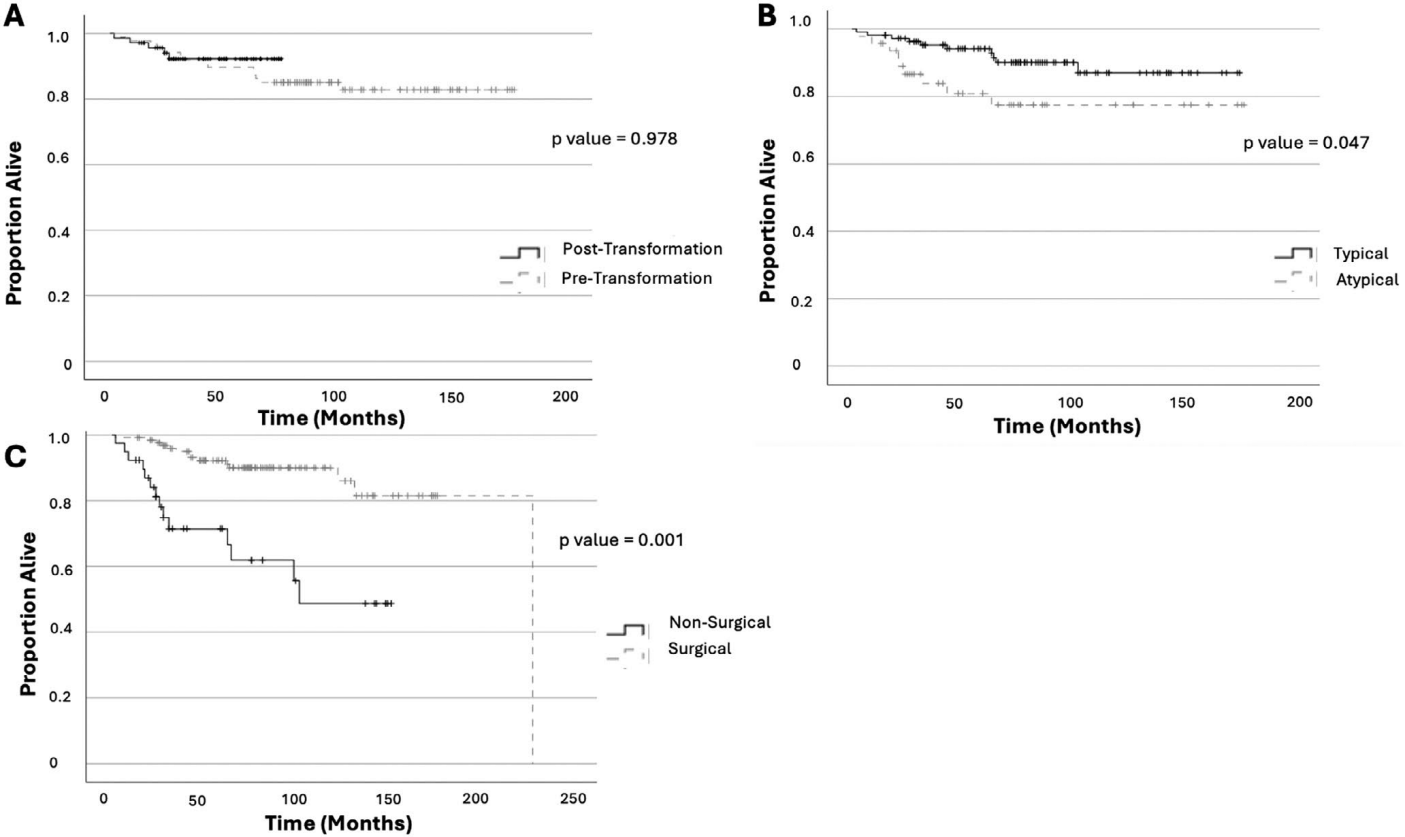
Kaplan–Meier survival curves (A) depicts the overall survival for pre‐ versus post‐transformation groups; (B) illustrate atypical versus typical carcinoids; (C) compare those having surgical resection versus nonsurgical therapies.

Kaplan–Meier survival curves for overall survival are shown in Figure 4 for pre‐ and post‐transformation groups, TC and AC, surgical and nonsurgical management groups. The median follow‐up times for both groups were 41 months (range: 2–128 months) in the pre‐transformation group and 40 months (range: 3–59 months) in the post‐transformation group. Due to much shorter follow‐up times for the more recent group, it was not possible to make a comparison although the KM curve suggests a trend for improved survival within 6 years. Patients with AC had a worse overall survival compared to those with TC (*p* < 0.05). A small number of patients (four) had a Ki‐67 index of > 20%, three were classed as AC and one TC.

## 4 Discussion

Bronchopulmonary NETs remain a relatively rare entity within the oncology landscape, accounting for a small fraction of all lung neoplasms but an important part of NEN MDT workload even if not as prominent as GEP‐NETs. We describe improvements in pathway times after transformation of our regional service encompassing all hospitals in the South Wales area. Although previous studies describe improvements in diagnostic modalities and treatments aligned with European guidelines, there is very little published data on service pathway times or suggestions for their improvement [11].

Diagnostic delays are an acknowledged challenge in bpNETs, often due to nonspecific symptomatology, although delays are not as profound as in GEP‐NETs. In our study, changes in service provision resulted in a statistically significant reduction in the time from presentation to diagnosis, symptom onset to diagnosis for symptomatic patients, and presentation to NET MDT discussion (*p* < 0.05). Furthermore, these improvements cannot be easily ascribed to other factors since there were no significant demographic or clinical differences between groups (gender, age, type of bpNET (TC or AC), Ki‐67, stage, functional status, or genetic syndromes). This contrasts with the literature, where median symptom‐to‐diagnosis times are reported as excessively prolonged, suggesting our service transformation may be a replicable model for other regions seeking to improve NET patient pathways [12, 13]. There is a paucity of studies evaluating pathway metrics in bpNETs but one study described a prolonged median symptom‐to‐diagnosis time of 53.8 months compared to 3–4 months in our study [14]. Robelin et al. described a much longer diagnosis to treatment time of 67 days (range 0–7595) compared to our 30 days although the previous study only included patients with metastatic disease, illustrating the difficulty in making comparisons [15].

Aligning with global incidence rates, our study revealed an incidence of TC and AC at 0.6 per 100 000 per year since 2017, at the lower end of the reported range (0.2–2 per 100 000 per year) in the literature [1]. This finding may reflect variances in reporting practices (we did not include neuroendocrine carcinomas), or true population‐level differences, suggesting a need for standardized, widespread epidemiological tracking to better ascertain true incidence rates [16]. The finding of more metastatic disease and worse overall survival in AC compared to TC is as expected given the more aggressive behavior of AC. The TC:AC ratio in our study was skewed toward TC (7:3), consistent with ratios observed in other case series [17, 18]. Only 3% of cases had pathogenic variants associated with MEN1 on genetic testing, which is consistent with frequencies identified in published series (1%–17%) [14].

When making diagnoses based on symptoms, clinicians need to remain vigilant of the poly‐symptomatology associated with NETs (Figure 2). Presence of cough (28%) was comparable to published data (14%–34%); however, our population reported more dyspnoea (20% vs. 12%) and less hemoptysis than published data in bpNETs (12% vs. 23%–32%) [14]. However, symptom frequencies are similar to other non‐NEN lung cancers [11]. Caution must be taken, however, when making direct comparisons due to heterogeneous populations and varying published symptom categories including “bronchial obstruction” [14]. Additionally, attribution of all symptoms to the underlying tumor is unclear. A small proportion of NETs in our study were functional, less than described in the literature (7.6%), emphasizing the message from NET centers that most symptoms in NENs are not caused by secretion of hormones [19].

Although the majority of cases presented with symptoms, a significant number of diagnoses emerged incidentally, a trend particularly noted among those with TCs, which is consistent with the more quiescent nature of TCs compared to ACs. Our incidental diagnosis rate at 17%–39% eclipses the reported rate in other studies (30%) [20]. However, our data were based on hospital records rather than patient recall and there was no rise in the proportion of incidental diagnoses since transformation. Therefore, the improvements in pathway times cannot be ascribed to rising asymptomatic presentations.

Following the service transformation, the increased usage of diagnostic assays (CgA, HIAA), advanced imaging techniques (Gallium PET), and central pathology review all reflect an alignment with contemporary international NEN guidelines through service transformation. This reflects the close collaboration between the central NET MDT and local respiratory teams. A rise in diagnostic tissue sampling and less reliance on surgical resection for histological diagnosis also represents improvements in adherence to guidelines, availability of a NET MDT, and availability of diagnostics. The fact that chromogranin A was not elevated in a significant subset of patients underscores the need for a nuanced understanding of tumor markers in the diagnostic process, and perhaps a reassessment of their utility and interpretation in clinical practice.

It is worth noting that Ki‐67 indices exceeding 20% were observed in some AC cases, suggesting a parallel phenomenon with Grade 3 well‐differentiated NETs arising in the gastrointestinal tract, commented in the recent literature where the importance of Ki‐67 is highlighted [21]. G3 gastroenteropancreatic NETs have a worse prognosis than Grade 1 or 2 NETs, but better than NECs. Although the incorporation of Ki‐67 into guidelines has been debated, this discovery raises questions about the current lung NEN classification systems which exclude Ki‐67 and whether they fully capture the spectrum of disease, underlining the complexity of NEN biology and the need for further studies of Ki‐67 in non‐gastroenteropancreatic NENs.

Our study suggests a paradigm shift toward more aggressive management, with less watchful waiting and more chemotherapy and targeted therapy, indicative of the NET multidisciplinary team's influence on treatment decisions. These findings align with broader trends in NEN management, which advocate for individualized, timely specialized interventions according to European guidance in NENs (ESMO and ENETS) [8, 10]. However, it is important to note that although much research focuses on systemic treatment of advanced and metastatic NENs, most cases have a surgical approach with surveillance after resection. Our study suggests survival is improved in this cohort. Although follow‐up guidance exists, the underlying evidence base is not strong and future research is required on this aspect.

A limitation in our study is not being able to assess times in primary care prior to referral. Despite a systematic approach to data collection, our study highlights the complexity of the diagnostic pathway. A key limitation of this study is the use of a historical control group spanning over two decades, which introduces potential confounding from changes in healthcare infrastructure, diagnostic technologies, and broader systemic changes unrelated to the NET service transformation. While we attempted to minimize bias by applying consistent data extraction methods and inclusion criteria across both cohorts, residual confounding by time cannot be excluded. A stratified or sensitivity analysis by 5‐ to 10‐year intervals was considered; however, this was not feasible due to small subgroup sizes and the uneven distribution of diagnoses across the 21‐year pre‐transformation period, which limited statistical power and comparability. However, symptom‐to‐presentation times appeared to change little and therefore improvements are unlikely to be explained by this less explored part of the pathway. Additional limitations include the retrospective nature of the study, which may be associated with missing or incomplete data, variability in documentation quality, and the absence of standardized clinical recording over time. As an observational study, randomization was not possible. The lack of overall survival analysis between groups due to the paucity of deaths within our study period is another limitation; however, fewer recurrences were noted in the post‐transformation group and there appears to be a trend toward improved overall survival. While promising, this must be interpreted cautiously due to the shorter follow‐up times and warrants extended monitoring to substantiate initial observations. As expected, survival for ACs was worse than TCs (and presence of metastases), and better for surgically resected cases (due to earlier curable disease), suggesting our population is comparable with other published series. While this care model was implemented within a devolved UK National Health Service (NHS) setting, its core principles, including earlier specialist input, central MDT review, and integrated diagnostic pathways, may be adaptable to other healthcare systems. However, generalizability may be influenced by variations in healthcare structure, resourcing, and policy. Finally, our approach of relying on hospital records, as opposed to patient surveys that make up the majority of publications in the area of NEN diagnostic pathways, serves to minimize recall bias and strengthens the validity of our findings [13, 14].

Despite the demonstrated improvements, the current service faces ongoing challenges, including increasing referral volumes, limited specialist workforce capacity, and the growing burden of long‐term follow‐up in a population with relatively indolent tumors. Scaling the model across wider regions requires careful planning and resource allocation. To support this, we have introduced a digital referral e‐form that centralizes submissions and enables automated triage, improving efficiency and reducing administrative burden. Current efforts include the publication of an all‐Wales follow‐up protocol that balances surveillance effectiveness with considerations around radiation exposure and patient longevity. Additionally, a prospective registry or audit tool is being explored to enhance data capture and support quality improvement. Emerging technologies such as AI‐driven triage or decision‐support tools may further streamline referral management and MDT coordination. Collectively, these initiatives aim to future‐proof the service and ensure sustainable, high‐quality care for patients with bronchopulmonary neuroendocrine tumors.

In conclusion, our study highlights the multifaceted improvements in bpNET diagnosis and management since the South Wales NET service transformation, one of the few studies to evaluate a regional service transformation in NETs. The expedited diagnosis and treatment times represent a significant advance in patient care which may be attributed to a service model change and closer relationships between NET MDTs and all local respiratory teams for this uncommon cancer. In the future, it will be essential to continue tracking metrics and outcomes to establish long‐term benefits of these service changes, ensuring that patient care is not only prompt but also impactful in improving survival and quality of life. Additionally, approaches to facilitate diagnosis of bpNETs at an earlier, and therefore, curable stage are required in addition to evidence‐based surveillance strategies.

## Author Contributions

**Mat Jones:** investigation, writing – review and editing. **Mohid S. Khan:** conceptualization, methodology, investigation, writing – original draft, writing – review and editing, formal analysis, supervision, project administration, data curation. **Helen E. Davies:** conceptualization, investigation, writing – review and editing, writing – original draft, formal analysis. **Arouba Imtiaz:** conceptualization, formal analysis, writing – original draft, methodology, writing – review and editing, data curation. **Oliver Burbidge:** investigation, writing – original draft, writing – review and editing, formal analysis. **Robin Ghosal:** investigation, writing – review and editing. **Janardhan Navaratnam:** conceptualization, methodology, writing – original draft, writing – review and editing, investigation, data curation, formal analysis. **Craig Dyer:** writing – review and editing, investigation, formal analysis.

## Funding

The authors have nothing to report.

## Ethics Statement

This study was conducted as a service evaluation within the NHS and therefore did not require formal Research Ethics Committee approval in accordance with guidance from the UK Health Research Authority. The project was registered as a service evaluation with Cardiff & Vale University Health Board and approved through the Health Board's Service Improvement governance process (registration: February 2022). Governance approval for data access and analysis was also obtained through the Health Board Information and Technology department. All data were extracted from routine clinical records and anonymized prior to analysis. No patient identifiable information was used. Cardiff & Vale University Health Board operates a patient data opt‐out policy in accordance with NHS data governance standards and Caldicott principles. Patients may opt out of their anonymized data being used for service evaluation and audit purposes.

## Consent

The authors have nothing to report.

## Conflicts of Interest

The authors declare no conflicts of interest.

## Data Availability

Research data are not shared.
